# Yoga as a complementary treatment for smoking cessation: rationale, study design and participant characteristics of the Quitting-in-Balance study

**DOI:** 10.1186/1472-6882-10-14

**Published:** 2010-04-29

**Authors:** Beth C Bock, Kathleen M Morrow, Bruce M Becker, David M Williams, Geoffrey Tremont, Ronnesia B Gaskins, Ernestine Jennings, Joseph Fava, Bess H Marcus

**Affiliations:** 1 Centers for Behavioral and Preventive Medicine, and Alpert School of Medicine at Brown University, The Miriam Hospital, Providence, RI 02903, USA; 2 Department of Emergency Medicine, Alpert School of Medicine at Brown University, Rhode Island Hospital, Providence, RI 02903, USA; 3 Department of Community Health, Alpert School of Medicine at Brown University, Providence, RI 02903, USA; 4 Department of Pediatrics, Alpert School of Medicine at Brown University, Women & Infants Hospital, Providence, RI 02903, USA

## Abstract

**Background:**

Tobacco smoking remains the leading preventable cause of death among American women. Exercise has shown promise as an aid to smoking cessation because it reduces weight gain and weight concerns, improves affect, and reduces nicotine withdrawal symptoms and cigarette craving. Studies have shown that the practice of yoga improves weight control, and reduces perceived stress and negative affect. Yoga practice also includes regulation of breathing and focused attention, both of which may enhance stress reduction and improve mood and well-being and may improve cessation outcomes.

**Methods/Design:**

This pilot efficacy study is designed to examine the rates of cessation among women randomized to either a novel, 8-week Yoga plus Cognitive Behavioral Therapy (CBT) smoking cessation intervention versus a Wellness program plus the same CBT smoking cessation intervention. Outcome measures include 7-day point prevalence abstinence at end of treatment, 3 and 6 months follow up and potential mediating variables (e.g., confidence in quitting smoking, self-efficacy). Other assessments include measures of mindfulness, spirituality, depressive symptoms, anxiety and perceived health (SF-36).

**Discussion:**

Innovative treatments are needed that address barriers to successful smoking cessation among men and women. The design chosen for this study will allow us to explore potential mediators of intervention efficacy so that we may better understand the mechanism(s) by which yoga may act as an effective complementary treatment for smoking cessation. If shown to be effective, yoga can offer an alternative to traditional exercise for reducing negative symptoms that often accompany smoking cessation and predict relapse to smoking among recent quitters.

**Trial Registration:**

ClinicalTrials NCT00492310

## Background

Over 17% of adult women in the United States are current smokers [1]. Tobacco smoking remains the leading preventable cause of death among American women and is a major cause of heart disease, lung and other cancers and many other illnesses [2]. Despite the importance of quitting, smokers encounter a number of significant barriers when they try to quit including nicotine addiction and its associated withdrawal symptoms, cigarette craving, increased negative affect, perceived stress, and post-cessation weight gain [3-6].

Aside from making quitting smoking uncomfortable, these symptoms also increase the individual's risk for relapse to smoking. Perceived stress, negative affect, and the severity of withdrawal symptoms have all been shown to predict time to relapse among recent ex-smokers [5-7]. Post-cessation weight gain and fear of weight gain are common among smokers. They often constitute a substantial barrier to initial cessation and are independent predictors of relapse to smoking, particularly among women [8-11]. Therapies that provide alternative methods to cope with withdrawal symptoms, negative affect, stress and concerns regarding weight gain should improve women's ability to quit and remain quit.

Exercise has been proposed as an aid to smoking cessation for women because of its ability to help offset weight gain during cessation and to reduce *concern *regarding weight gain [12,13]. Because exercise improves affect in adult populations [14-20], exercise may also aid cessation by reducing negative affect and withdrawal symptoms [21,12]. Previous studies have demonstrated that a traditional aerobic exercise program (i.e., brisk walking, bicycling) combined with Cognitive-Behavioral Therapy (CBT) produces higher quit rates among women at post-treatment and six-month follow up compared to CBT without exercise [12,22].

Yoga is an alternative to traditional (western) exercise that has potential as a complementary treatment for smoking cessation. Yoga is comprised of several elements including regulation of breathing, *asanas *(yoga postures), and focused attention (e.g., meditation), all of which may enhance stress reduction and improve mood and well-being [23], which are linked to successful smoking cessation [24]. Several studies have shown that the practice of yoga improves weight control [25-28], and reduces perceived stress and negative affect [29-32]. Based on a synthesis of this evidence-based research, this study is designed to examine the effects of an 8-week yoga program on perceived stress, affect, and the process of quitting smoking among healthy, adult women smokers.

## Methods/Design

### Design

The study design is a randomized, controlled pilot study comparing two conditions: 1) group-based cognitive-behavioral therapy (CBT) for smoking cessation plus a twice-weekly group-based yoga program (***Yoga***); and 2) CBT for smoking cessation plus a group-based wellness program that serves as a contact-control (***Wellness***). Groups of 8-10 individuals enter the program in sequential cohorts and participate in the 8-week treatment program. Assessments take place at baseline (before program entry), end-of-treatment (week 8), and both 3 and 6 months follow up.

### Participant recruitment

Recruitment methods include placing advertisements in local newspapers and websites, posting flyers and placing brochures at commercial locations (e.g., convenience stores, grocery stores) and physician's offices, and television coverage. The study research assistant (RA) is trained to provide information about the program to women who call the program in response to the advertisements, and to screen interested persons for eligibility. Callers are excluded if they smoke less than 5 cigarettes per day, are already physically active (> 3 days of moderate-intensity physical activity or >2 days vigorous intensity exercise per week); are currently practicing yoga, have current heart disease, lung disease, or orthopedic conditions that could make participation in yoga difficult or dangerous; or are currently in treatment for psychiatric illness or are using illegal substances. Eligible participants are also screened to ensure safe participation in physical activity by using the PAR-Q [33], and are sent a letter for their physician to sign giving permission to participate in the program. Individuals deemed eligible following screening are then scheduled for an introductory orientation session during which the study RA presents the details of the study, reviews what is required of participants and administers informed consent. All recruitment procedures and materials including advertisements, consent forms and procedures are approved by the Miriam Hospital Clinical Research Review Board (IRB registration # 0000482).

### Procedures

Enrolled participants are randomly assigned to either the yoga or wellness arms of the study. All participants are also provided on a weekly basis with 1-hour of group based CBT for smoking cessation (separate groups are held for yoga and wellness participants). One yoga and one wellness session per week are scheduled for the same night as the smoking group treatment, so that participants need to travel to the program only two evenings per week.

### Interventions

**Figure 1:**
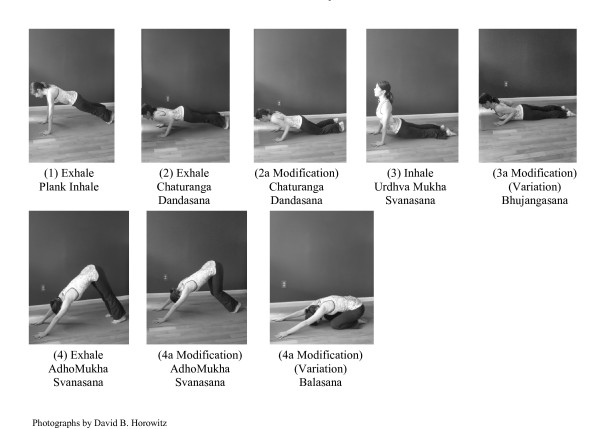
**Basic Vinyasa Yoga Asanas**.

Yoga classes are conducted twice weekly for eight consecutive weeks by certified yoga instructors with over 15 years experience and who are trained in the *Vinyasa *style. *Vinyasa *is a form of Hatha yoga that emphasizes the continuous flow of movement between postures (*asanas*) and links breath with the movements. In collaboration with the researchers, yoga instructors select a pattern of *asanas *that are appropriate for beginners and teach them consistently during each class. Each class consists of five minutes of breathing exercises (*pranayama*) and seated meditation, followed by 45 minutes of dynamically linked *asanas *(see Figure 1). Classes conclude with ten minutes of closing postures (similar to the "cooling down" period following aerobic exercise) and a final seated meditation.

Participants attending the wellness sessions are shown videos regarding a variety of health topics (e.g., low fat diets, breast cancer screenings, back health) followed by a discussion guided by the study RA and distribution of written material relevant to the video topic.

The CBT smoking cessation program is lead by a PhD psychologist with over 10 years experience in conducting smoking cessation groups. Treatment is delivered through use of a program manual to ensure consistent delivery of program content. Topics include self-monitoring, stimulus control, coping with high-risk situations, and stress management for smoking cessation. The program also focuses on topics of concern to women when quitting including: healthy eating, weight management, and balancing multiple roles and multiple demands. This program has been used in our prior studies [12,22]. Participants are also encouraged to select healthy, low-calorie snacks throughout the program and are provided with examples of these snacks to sample. Participants are not provided with nicotine replacement therapy (NRT) or other smoking cessation medications, but are allowed to use them in conjunction with the program, if their primary care physician deems it advisable.

### Measures

Participants complete assessments at baseline, end of treatment (week 8), and both 3 and 6 month follow up (see Table 1). Assessment instruments include surveys of demographic variables, smoking history and nicotine dependence [34] (FTND), Smoking Situations Temptations scale [35](SST), Stage of Change for Smoking Cessation [36], the Smoking Self-efficacy scale [35] (SSE), Withdrawal Symptoms Checklist [37]; and a single item assessing confidence for achieving smoking cessation. Participants also complete the Freiberg Mindfulness Inventory [38] (FMI), and measures of spirituality [39] (STI); [40,41] Anxiety (STAIT); and depressive symptoms [42](CESD-10). Participants also complete assessments of exercise participation [43,44] (PAR), Physical Self-Perception Profile [45] (PSPP); the Short-Form Health Survey [46] (SF-36), and assessments of concern regarding post-cessation weight gain [47] (SSQ).

**Table 1:** Measurement Schedule

Measure	Baseline	weeks 1-8	Post- treatment	Month 3	Month 6
Demographics	✘				
**Smoking Status**					
Smoking History	✘				
Nicotine dependence [34]					
Smoking Behavior, Carbon monoxide	✘	✘	✘	✘	✘
Saliva Cotinine [49]			✘	✘	✘
Withdrawal Symptoms,[37]	✘	✘	✘	✘	✘
**Physical Activity Status**					
7-Day PAR [43,44]	✘		✘	✘	✘
PARQ [33]	✘				
**Weight concerns**					
Weight Concerns [47]	✘		✘	✘	✘
**Psychosocial Measures**					
Self-efficacy, Stage of Change [35,36]	✘		✘	✘	✘
Mindfulness [38]	✘		✘	✘	✘
Spirituality [39]					
Depression [42]	✘		✘	✘	✘
Anxiety [40,41]					
Physical Self-perception [45]	✘		✘	✘	✘
Short form health survey [46]					
Mood (PANAS) [48]		✘			
Cigarette craving					
**Recruitment, Retention and Satisfaction Measures**					
Attendance and drop out		✘	✘		
Group Cohesion			✘		
Consumer Satisfaction			✘		

Each week during the program, participants are assessed for current smoking status (verified by exhaled carbon monoxide [CO]). To assess for acute changes, current positive and negative affect [48] (PANAS), nicotine withdrawal symptoms and cigarette craving are measured once weekly pre- and post-session (yoga or wellness). Smoking outcomes (7-day point prevalence abstinence) are biochemically validated by exhaled carbon monoxide (cutoff < 10 ppm) and saliva cotinine (cutoff = 15 mg/ml)[49].

At the end of each cohort, separate focus groups are to be held for the yoga and wellness participants. Focus groups are conducted by a PhD Psychologist with over 10 years experience in leading focus groups and conducting qualitative research. Focus groups discuss acceptability of the study protocols and intervention components. In particular, discussions explore participant opinions and observations regarding the intervention content, program format, and study design (e.g., program length, timing of sessions, and the number of classes per week). Each of these questions is addressed primarily from the standpoint of the individual participant's experience with the program. A qualitative data collection guide was prepared by the investigators to delineate topics and subtopics to be addressed during the focus group discussions. Each focus group requires approximately 2 hours to be completed, and is audio taped and professionally transcribed for analysis.

### Outcomes

The primary outcomes for this study are post-treatment cessation rates, study feasibility and acceptability. Differences in post-treatment (12 week) cessation rates between conditions will provide data for estimates of effect size necessary to calculate power estimates for a major clinical trial. The qualitative and quantitative feasibility and acceptability of the Quitting in Balance program will be assessed by the following outcome measures at the individual participant level:

1. Smoking abstinence rates

2. Accrual and Retention rates

3. Acceptance of Randomization

4. Compliance with study protocol

The feasibility and acceptability outcome measures listed above will be assessed at baseline, 12-weeks (end of treatment), and 3 and 6 months follow-up.

### Sample size

The nature of this innovative exploratory treatment study necessarily provides limits on the effective sample size, limiting the number of smokers that can be recruited, complete instruments, and participate in the intervention. There is not yet sufficient information to estimate a potential treatment effect size for the CBT + Yoga condition. Thus, our primary interest was to determine a valid estimate of the yoga intervention effect size for smoking cessation for a future, larger efficacy trial. Consequently, we did not conduct power analysis calculations. Rather, the required sample size was based on the number of participants needed to perform valid estimates of the CBT + Yoga intervention smoking cessation rate and possible differential smoking cessation rates between treatments. Given the typical differences in quit rates in most smoking cessation studies, often 10%-15%, [12,22,48,50], we determined that a sample size of 60 participants would be sufficient to obtain initial estimates of effect size with 60% power (medium effect size).

### Randomization

After attending an initial Orientation session, in which the study and its requirements will be explained and participants have provided written consent, participants will be randomly assigned to one of the two investigational conditions. Randomization will be conducted the first evening of the program with all participants present using a simple randomization protocol.

### Blinding

Because of the nature of the study, it is not possible to blind participants or the study counselors to condition. Smoking cessation therapy will be offered in a group setting during which time participants often remark on the content of the yoga or wellness program. Research assistants who conduct all assessments will be blind to study condition.

### Implementation

Adult women smokers who are seeking treatment for quitting smoking will be recruited through advertisements in local newspapers, flyers at grocery stores and Internet advertisements. Participants will be included in the trial if they meet inclusion criteria and have signed informed consent. To ensure the implementation of the intervention, quality audits will be conducted. A PhD clinician will listen to audio tapes of 50% of all group CBT sessions and use an objective ratings sheet to record the quality of coverage for all planned topics listed in the treatment manual.

### Time Plan

The development of this program began in August of 2007 and was finished in December 2007. Participant recruitment started in January, 2008 and continued through May 2009. The trial will be completed in June 2010.

### Planned Analyses

The primary outcome variable is quit status, verified with carbon monoxide (cutoff, 8 ppm) weekly, and with saliva cotinine (cutoff, 15 ng/ml) at the end of treatment, and both 3 and 6 month follow up assessments [49]. To be considered abstinent, subjects need to have a carbon monoxide level less than 8 ppm and a cotinine level less than 57 nmol/L [15 ng/mL].

The primary hypothesis of the study will be tested using a logistic regression to produce an odds ratio measuring the effect of CBT + Yoga on the likelihood of smoking cessation at the post-treatment and follow up assessments. The intention-to-treat principle will be used to calculate treatment effects and differences using all participants initially assigned to treatment. Analyses will also include calculations of the proportion of participants who are continuously abstinent, and abstinent as assessed for 7-day point-prevalence for both treatment groups in order to provide a fuller estimate and understanding of the possible range of the treatment effects. In addition, we will conduct a second longitudinal analysis across time (end of treatment, 3 and 6 month follow up after treatment) using the generalized estimating equations (GEE) approach of Zeger and Liang [51]. This procedure can accommodate covariates and missing data, thus allowing the maximum use of existing data (and thus enhanced power) for detecting treatment effects. This approach is also highly flexible for modeling trend analyses that allow for an alternate way to present intervention effectiveness (comparing trends in both groups over time).

Secondary analyses will examine the effects of yoga on other psychological and behavioral domains including anxiety, quality of life, spirituality, mindfulness and self-esteem. These analyses will consist of a series of repeated measures analyses of covariance using the baseline measure of each variable as a covariate and 3 repeated measurements (end of treatment, 3 and 6 month follow up).

Further analyses will examine the effects of the CBT + Yoga intervention on potential mediators of smoking behavior change, and the relationships of the potential mediators to smoking cessation. The potential mediators of interest include changes in affect, perceived stress, well being, and self-efficacy as well as cigarette craving and withdrawal symptoms. We will also examine for any differences in the effects of the CBT + Yoga intervention versus the CBT + Wellness intervention on these intermediate variables. Given the limited sample size in the proposed study, these analyses will be exploratory in nature, and within the context of these analyses, we will be trying to establish 2 of the 3 key directional effects needed to describe potential mediators as noted by Baron and Kenny [52], that is we will be investigating which of the potential mediating variables are affected by the treatment, and which of these potential mediators are also related to the key smoking cessation outcome variables. The strongest evidence for mediation is time-ordered, and these analyses will examine the effects of treatment on each of the various potential mediating variables at immediate post-treatment (8 week assessment), and include analyses of possible longitudinal treatment effects extending to the 3 and 6 month follow up. These analyses will consist of a series of repeated measures analyses of covariance using the baseline measure of each potential mediator variable as a covariate and 3 repeated measurements (end of treatment, 3 and 6 month follow up). We will conduct these analyses using Proc MIXED in SAS, which will allow us to easily conduct contrast tests to compare both between and within group effects across time. Proc MIXED also accommodates missing values under the assumption of missing at random [53], and will allow us to make maximum use of the existing data to detect treatment effects. To examine the effects of potential mediator variables on treatment outcome we will conduct a series of time-ordered logistic regressions, (e.g., post-treatment value of smoking self-efficacy, perceived stress and well-being, etc. predicting both continuous abstinence and 7-day point prevalence at 3-month follow-up).

Lastly, we will conduct qualitative analysis of the focus group material for common and emergent themes emphasizing aspects of the program that participants liked most and least, along with their suggestions for changes. Data will be coded by themes derived from the focus group guide and managed using NVivo qualitative data software. The range of participant opinions, experiences and observations will be summarized and interpreted as feedback for consideration in revising future iterations of the intervention.

### Analyses of Baseline Data

The baseline demographic, psychosocial, and smoking-related characteristics of the study participants are described here using means, standard deviations, range values and proportions. Stage of Change for Smoking Cessation is calculated in two ways, with the first based solely on intention to quit, while the second classifies participants into the Preparation stage if they both intend to quit in the next 30 days and they have made at least 1 quit attempt (≥24 hr) in the past year. To describe participant's experience with withdrawal symptoms during previous quit attempts we calculated the percentage of the sample that endorsed 3 or higher on the Withdrawal Symptoms checklist [37] (scored from 1 = "not at all' to 5 = "extremely"), which assessed the severity of nicotine withdrawal symptoms.

## Discussion

If shown to be effective, yoga can offer an alternative to traditional exercise for reducing negative symptoms that often accompany smoking cessation and predict relapse to smoking among recent quitters. Yoga may enhance the effects of CBT by providing an alternate method for coping with stress and cigarette cravings, and may be associated with improvements in mood and quality of life. Yoga is intended to be a lifelong practice that can potentially reinforce quitting smoking once formal smoking treatment ends.

In addition to making quitting smoking uncomfortable, symptoms including perceived stress, negative affect and the overall severity of withdrawal symptoms have all been shown to increase the individual's risk for relapse to smoking. Relapse to smoking within the first week of quitting is preceded by increases in withdrawal symptoms, including craving for cigarettes, distress, and reductions in positive affect [54]. Perceived stress and negative affect have also been shown to predict time to relapse among recent ex-smokers [5,6,55,56]. Providing smokers with methods of coping with stress should improve their ability to quit and remain quit [57].

Several studies have documented that traditional (western) aerobic exercise improves smoking cessation among women [12,22,58]. Vinyasa yoga, which includes both aerobic exercise as well as other features such as focused attention and deliberate regulation of breathing, may be especially beneficial to women who are trying to quit smoking. The goal of this study is to examine the utility of a program of yoga as a complementary treatment to cognitive-behavioral therapy for smoking cessation.

This study is an initial investigation of the efficacy of yoga as a complementary treatment for smoking cessation among women. While our prior studies used a 12-week treatment protocol to ensure that changes in physical fitness could be measured, we chose to deliver treatment over 8 weeks. This choice was made for several reasons. First, the effects of exercise on mood and cigarette craving seen in our previous study [21] were acute, rather than chronic-training effects. Thus, it seems likely that the effects of yoga practice on these symptoms would also be produced acutely. The 12-week protocol used in our other studies was chosen to allow sufficient time for fitness changes to take place. Since we were not hypothesizing that fitness changes were responsible for the effect of yoga on smoking cessation, measuring a training effect was deemed unnecessary. Second, the use of a shorter overall treatment program reduces subject burden and is likely to lower the risk of dropout. Third, participants in our previous studies were very unlikely to make an initial quit after week 8, therefore extending CBT beyond week 8 seemed unlikely to increase treatment effectiveness. Another design choice made in developing this study was the use of a Health & Wellness program as a comparison condition. An alternative design would have been to test CBT alone (with no adjunctive program) compared to CBT plus yoga. In some ways, the second option would have provided a more "real world" comparison, in that the use of yoga with CBT does add additional time and treatment burden to quitting smoking. However, we chose to use the Healthy & Wellness program to control for the amount of time participants were in contact with the research staff (contact-control) and to equalize subject burden. Last, because this is an initial study of the potential for using yoga as a complementary therapy in smoking cessation, we also chose to use a variety of measures of affect, self-perception and experiential variables (e.g., FMI assessment of mindfulness). The design chosen for this study will allow us to explore potential mediators of intervention efficacy so that we may better understand the mechanism(s) by which yoga may act as an effective complementary treatment for smoking cessation.

Innovative treatments are needed that address barriers to successful smoking cessation among men and women. The practice of yoga has been linked to improvements in affect, reductions in stress and anxiety, increases in self-efficacy and improvements in overall well-being in addition to producing improvements in cardiopulmonary functioning [29-32]. Therefore, yoga may prove to be a more effective complementary treatment for smoking cessation than traditional aerobic exercise.

## Competing interests

The authors declare that they have no competing interests.

## Authors' contributions

BCB, BMB and BM conceived of the study and participated in its design and coordination. JF conducted the data analyses. KM, DW, RG, EJ and BM assisted with the development of research protocols. All authors read and approved the final manuscript.

## Authors' information

BCB is an Associate Professor in the Department of Psychiatry and Human Behavior at Brown Alpert Medical with over 15 years experience with conducting and implementing randomized control trials for smoking cessation and exercise intervention among adults.

## Pre-publication history

The pre-publication history for this paper can be accessed here:

http://www.biomedcentral.com/1472-6882/10/14/prepub
